# Gallium nitride micro-light-emitting diode structured light sources for multi-modal optical wireless communications systems

**DOI:** 10.1098/rsta.2019.0185

**Published:** 2020-03-02

**Authors:** A. D. Griffiths, J. Herrnsdorf, J. J. D. McKendry, M. J. Strain, M. D. Dawson

**Affiliations:** Institute of Photonics, Department of Physics, University of Strathclyde, Glasgow G1 1RD, UK

**Keywords:** light-emitting diodes, Gallium nitride, structured light, optical wireless communications

## Abstract

Gallium nitride-based light-emitting diodes (LEDs) have revolutionized the lighting industry with their efficient generation of blue and green light. While broad-area (square millimetre) devices have become the dominant LED lighting technology, fabricating LEDs into micro-scale pixels (micro-LEDs) yields further advantages for optical wireless communications (OWC), and for the development of smart-lighting applications such as tracking and imaging. The smaller active areas of micro-LEDs result in high current density operation, providing high modulation bandwidths and increased optical power density. Fabricating micro-LEDs in array formats allows device layouts to be tailored for target applications and provides additional degrees of freedom for OWC systems. Temporal and spatial control is crucial to use the full potential of these micro-scale sources, and is achieved by bonding arrays to pitch-matched complementary metal-oxide-semiconductor control electronics. These compact, integrated chips operate as digital-to-light converters, providing optical signals from digital inputs. Applying the devices as projection systems allows structured light patterns to be used for tracking and self-location, while simultaneously providing space-division multiple access communication links. The high-speed nature of micro-LED array devices, combined with spatial and temporal control, allows many modes of operation for OWC providing complex functionality with chip-scale devices.

This article is part of the theme issue ‘Optical wireless communication’.

## 1. Introduction

The wireless transmission of data is ubiquitous in the modern world, with both the number of devices and volume of data transfer ever increasing to support a wirelessly connected Internet of Things (IoT). The dominant technology for wireless communications is currently radio frequency (RF), though it is expected that RF capacity will be unable to keep pace with the current increases in demand [1]. It is, therefore, unlikely that future wireless communication will be dominated by a single technology. Optical wireless communications (OWC) involves data transmission using optical frequencies (ultraviolet, visible and infrared), and is an attractive, complementary approach which may ease the capacity strain on RF communications. Moving to the optical spectrum has a number of benefits, including: wide unlicensed bandwidth, no interference with RF signals, and spatial reuse and security through signal confinement [2]. In the more specific case of light-emitting diode (LED) transmitters, advantages also include energy efficiency, low cost, size, weight and power, and the potential for integration into current solid-state lighting (SSL) for ‘Li-Fi’ (light-fidelity) style systems [3].

The source technologies that underpin visible light OWC include Gallium nitride (GaN) LEDs [4], III-Phosphide LEDs [5], organic/colloidal quantum dot/perovskite LEDs [6–8] and visible light semiconductor lasers [9]. Among these, GaN LEDs have seen a high level of research activity because they offer a combination of robustness, high performance, technological simplicity, maturity and versatile device formats with routes to scalable manufacture. A format that offers a particularly rich array of opportunities is microscale GaN LEDs with a size of less than 100 *μ*m, referred to as ‘micro-LEDs’. Individual micro-LEDs have intriguing properties on their own, as their dimensions enable unique operating conditions such as high current densities. This, in turn, allows high modulation bandwidths of 100s of MHz, making these devices attractive for OWC (see §2). Notably, micro-LEDs are typically operated as arrays, adding a spatial degree of freedom to the LED modulation and significantly extending their applications beyond the capabilities of conventional broad area LEDs. Importantly, micro-LEDs are also emerging as a new format of high brightness and fast response display technology, therefore offering routes to convergence between lighting, display and communication technologies.

This article will discuss the particular characteristics of micro-LEDs, the challenges in interfacing and driving high-density arrays, and the application areas opened up by the devices that have been developed. In §2, the characteristics of micro-LED pixels are discussed, while §3 covers driving and interfacing methods for high-density micro-LED arrays. In §4, recent work on OWC with micro-LED sources is presented. Section 5 discusses multi-functional OWC enabled by the structured light projection from micro-LED devices. Finally, §6 draws some conclusions and provides an outlook on future work.

## 2. Characteristics of micro-light-emitting diodes for optical wireless communication

LEDs are increasingly widespread devices used in a broad range of applications, currently revolutionizing indoor and street lighting [5], and set to play an increasingly important role in display technology. The III-V compound semiconductor alloys can provide devices with emission wavelengths across the full visible spectrum, extending into the ultraviolet and infrared. With efficient blue-emitting devices available from GaN-based materials, white light can be generated with the addition of a suitable phosphorescent/fluorescent layer, or in combination with other LED sources emitting at longer wavelengths, enabling SSL [10]. The advantages of LED-based lighting over other previous lighting technologies, particularly in efficiency and operational lifetime, are now well known. However, the semiconductor nature of LEDs offers ready interfacing to advanced electronics and allows applications to be investigated far beyond simple illumination. A prominent example is Li-Fi technology, which can use either broad area or micro-LEDs, though the micro-LED format enables a range of capabilities that are largely inaccessible to broad area LEDs.

Micro-pixellated arrays of inorganic LEDs can also be used as passive or active-matrix displays, which offer high brightness compared to existing liquid crystal or organic LED technologies [11,12], can be readily combined with nanocrystal colour converters for a large colour gamut [13], and are likely to play a significant role in emerging display technologies. For such an application, devices will need to provide around 100 fps refresh rates over a great number of pixels. However, each pixel in an array has a fast modulation response, and while backplane electronics with matching response will be difficult to achieve for high pixel numbers, smaller scale arrays are attractive for OWC using the schemes discussed in §4 and 5. The exact properties of the micro-LEDs depend on the device format, electronic interfacing, and driving conditions.

### (a) Fabrication and format

Inorganic LEDs are fabricated from wafers with epitaxially deposited semiconductor films, which include p-doped and n-doped layers, a multi-quantum-well junction region, and possibly other functional layers such as an electron blocking layer [14]. GaN-based epitaxial structures are commonly grown on sapphire substrates, though silicon and bulk GaN wafers are also in use [15]. The use of transparent substrates in particular, such as sapphire, enables a flip-chip device format which is crucially exploited in flip-chip active matrix devices [16,17].

**Figure 1.**
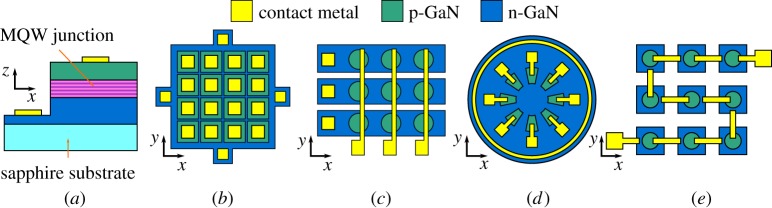
Schematics of micro-LED structure and array formats (not to scale): (*a*) side view of a pixel’s vertical structure and plan views of (*b*) active-matrix array, (*c*) passive-matrix array, (*d*) low-density array, (*e*) series connected array. (Online version in colour.)

Individual LED pixels are fabricated by etching mesas into the epitaxial structure of the wafer, isolating an area of the active region and allowing access to both the n-doped and p-doped regions for electrical contact. Mesas are defined with photolithographic patterning followed by inductively coupled plasma (ICP) etching and contacted through deposition of metal tracks and pads, resulting in the structure shown in the schematic cross section in figure 1*a*. Various pixel arrangements can be defined through design of the photolithography masks, allowing micro-LED arrays to be tailored for specific applications. High-density active- or passive-matrix arrays (figure 1*b*,*c*, respectively) were originally designed for display applications, but are now also used for structured illumination, digital-to-light conversion and other related techniques. Low-density arrays of individually connected micro-LEDs (figure 1*d*) have proven useful for high data rate multiple-input multiple-output (MIMO) OWC. Micro-LED arrays without pixel-addressing capability, e.g. through series connection (figure 1*e*), have also shown attractive properties [18]. Sections §4 and 5 highlight how these device formats relate to particular applications.

### (b) Active-matrix arrays

Early generations of LED displays such as alphanumeric indicators and dot-matrix displays were fabricated in a passive-matrix format, requiring continuous line-scanning for the display of arbitrary patterns. While impressive demonstrations are still being made with such devices today, including e.g. a 1000 fps single-pixel camera [19], there are clear scalability issues, with traditional displays starting to experience flicker-issues when going beyond 0.01 Megapixels [20]. Addressing pixels in an array with direct, individual contacts, with separate electronic drivers, is even more limiting as the space requirements of the metal tracks make it difficult to create arrays larger than 10 × 10 [21].

**Figure 2.**
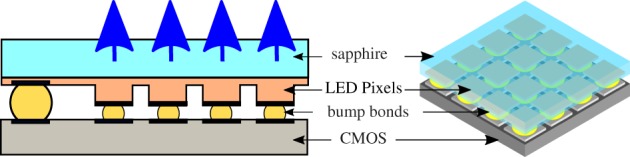
Schematic of a micro-LED array bump-bonded to a CMOS control chip. The flip-chip format of the array means light is extracted through the sapphire substrate, which is polished. (Online version in colour.)

Therefore, an important advance was made by the introduction of active-matrix arrays, where each micro-LED pixel has its own driver circuit, which in turn is controlled through a digital electronic bus. One approach is to develop monolithic devices, with both LED pixels and transistors fabricated in a single GaN-based epitaxial structure [22]. However, this approach has several challenges including the complexity of the required epitaxial structure and difficulties in obtaining a high area fill factor. Instead, digital drivers can be readily implemented in complementary metal-oxide-semiconductor (CMOS) control electronics, in an arrayed format matching that of a micro-LED array. Vertically stacking the micro-LED array on top of the CMOS control array, using flip-chip bump bonding, provides a millimetre-scale chip with digital control of optical emission. Each LED pixel has an individual set of drive electronics and light is extracted through the optically transparent sapphire substrate as shown in figure 2. Complex digital drive electronics can be implemented, allowing additional functionality for an active-matrix micro-LED system such as pulse width modulation (PWM), memory buffers for image display and short pulse operation [16,23]. Additionally, more complex, analogue modulation can be performed using an integrated driving approach by developing CMOS electronics with a current-steering digital-to-analogue converter (DAC) to drive the micro-LED pixels [24]. Besides flip-chip bonding, integration of GaN micro-LEDs with CMOS drivers can also be achieved through transfer printing individual LED pixels from the growth substrate onto the CMOS chip [25]. This approach produces devices with high uniformity and low optical cross-talk, though the process has a low level of maturity.

### (c) Modulation characteristics

**Figure 3.**
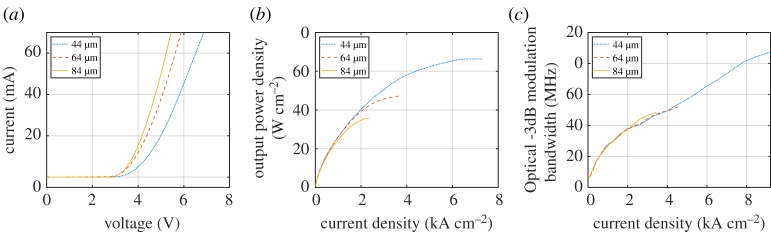
Example (*a*) I–V, (*b*) L–I and (*c*) modulation bandwidth characteristics of circular, blue (450 nm) micro-LED pixels with varying diameters [26]. Output power density was measured externally at the sapphire face. (Online version in colour.)

LED performance under DC operation is characterized by the relationships between bias voltage, current draw and output optical power. Figure 3*a* shows the current in a micro-LED pixel as a function of bias voltage. An immediately clear feature is the very high current density operation in micro-LEDs, in excess of 1 kA cm^−2^, compared to typical broad area devices operating around 100 A cm^−2^. Figure 3*b* shows the output power density, dependent on the current density, displaying the high brightness performance of micro-LED pixels. The nonlinear relationships between current, voltage and output optical power shown have important consequences in the modulated driving of micro-LEDs, and this is discussed in detail in §3.

To transmit OWC signals, the micro-LED transmitter must emit a modulated optical signal. Output intensity modulation is achieved by applying a modulated electrical signal in combination with DC bias conditions, but there will ultimately be a limit to how rapidly a device can respond to the input signal. An important parameter of a device is, therefore, the optical modulation bandwidth that can be achieved, defined as the frequency at which the power of the optical signal is reduced to half, or −3 dB. On an electrical frequency response curve from a photodetector, this corresponds to the −6 dB point, as received electrical power is proportional to the square of the photocurrent generated by incident optical power. Figure 3*c* shows the modulation bandwidth of a range of micro-LED pixels versus increasing current density. This bandwidth was measured by applying a DC-offset small-signal sinusoidal signal. Increasing the DC bias, and therefore injected current density, increases the modulation bandwidth.

For a broad area LED, the modulation bandwidth is typically limited by the RC (resistance—capacitance) constant of the device, which is on the order of nanoseconds for devices with an area on the order of 1 mm^2^. By contrast, for a 100 × 100 *μ*m micro-LED, the RC constant is estimated to be on the order of 200 ps [27], and thus for such small devices, other parameters limit the bandwidth. When comparing the bandwidths of micro-LEDs with different sizes (figure 3*c*), we observe the same bandwidth at a given current density regardless of the micro-LED area. This indicates that the bandwidth is determined by the differential carrier lifetime in the active region [26]. This can be understood by the current-density dependence of carrier lifetimes in semiconductors, which are often approximated by the popular ABC model [28]. An increase in current density results in an increase in the carrier density within the active region, which in turn results in increased radiative and higher-order (Auger) recombination rates, directly improving modulation bandwidth [29]. It should be noted that the device physics of GaN LEDs are notoriously complicated and both RC constant estimates and ABC model analysis need to be interpreted with care [27,30]. However, they are useful to understand the dominating factors affecting the modulation characteristics.

## 3. Driving modes and electronic interfacing

### (a) Individual pixel driving

**Figure 4.**
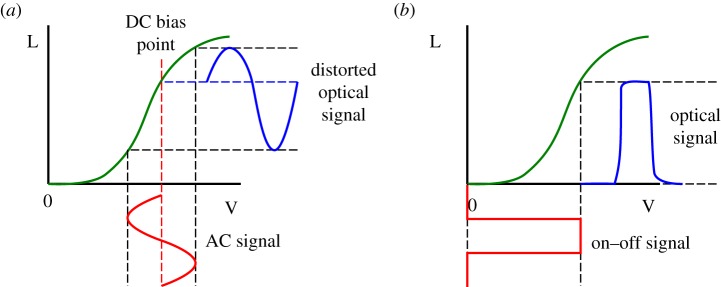
Schematics of (*a*) modulation of an LED with a DC-biased AC signal. Input electrical signals (red) can be distorted in the optical output (blue) by the nonlinear characteristics of an LED (green). (*b*) Modulation by on–off switching. (Online version in colour.)

Intensity modulation of an LED transmitter is typically performed by applying an AC signal to the applied driving voltage. The modulated voltage must remain positive and above the turn-on value for the LED to emit light, so the AC signal is often applied with a DC bias. Such an approach allows complex modulation schemes to be employed in the AC signal, such as pulse amplitude modulation (PAM) and orthogonal frequency division multiplexing (OFDM), and is often used for high data rate, single transmitter demonstrations [4]. However, LED luminosity is nonlinearly dependent on the current flow through the active region of a device, so the relationship between the electrical driving signal and the output optical signal is highly nonlinear. Figure 4*a* shows this schematically for a voltage signal. This can have significant impact on complex modulation schemes as the signal becomes distorted, and care must be taken to operate in the quasi-linear region of the luminosity–voltage (L–V), or luminosity–current curve (L–I), restricting the dynamic range of an OWC system.

DC-biased AC signals work well for single micro-LED transmitters, as the emitter can be wire-bonded to a printed circuit board (PCB) where arbitrary electrical signals can be applied by a suitable external driver, typically an arbitrary wave-form generator (AWG) or a DAC. This approach can yield high data rates with low-density arrays of micro-LEDs [31,32], however, there are obvious scaling problems for addressing high-density arrays at rates producing Mb s^−1^–Gb s^−1^ data signals typically expected of OWC.

Therefore, a common and much simpler approach is full on/off switching of the LED pixels as indicated in figure 4*b*. This is typically realized through p-type MOS (PMOS) or n-type MOS (NMOS) field-effect transistors (FETs) connected to the anode/cathode of the LEDs, and thus readily implemented in high-density active matrix arrays. While NMOS transistors provide higher switching speeds and lower area requirements than PMOS transistors, they need to be connected to the LED cathodes. Standard GaN epitaxial structures have the p-type material on top, due to the low conductivity of p-type GaN, thus imposing significant fabrication challenges when using NMOS drivers, including etching down to the sapphire substrate and depositing several insulation layers [33]. Therefore, NMOS drivers are only used in a few select cases where fast switching speeds were desired, and PMOS drivers are much more commonly used.

Using such on/off switching, micro-LED pixels can also (when required) be driven by extremely short electrical pulses, with frequency components beyond the modulation bandwidth of the device. This has produced optical pulses as short as 300 ps with an output power density of ≈10 W cm^−2^ [16].

### (b) Highly parallel digital electronic interfacing

As discussed above in §3a, micro-LED-based OWC systems will in practice rely on driving through either DACs or FETs, both of which can be directly controlled through digital electronic signals. These digital control signals in turn are supplied through application-specific integrated circuits (ASICs), micro-controllers, or through field-programmable gate arrays (FPGAs). All of these provide a low footprint in terms of size, weight and power (SWaP), thus retaining this important property of micro-LEDs, and they can connect to standard computer interfaces such as universal serial bus.

Micro-controllers are convenient and sometimes used for proof-of-principle demonstrations, but their performance does not match the capabilities of micro-LED systems. ASICs are very powerful, but their development is time-consuming and costly, so they are only used in research if there is no simpler method to achieve the operating target. FPGAs are more difficult to use than micro-controllers, but they offer comparable performance to ASICs and allow exact control of latencies. Consequently, FPGAs have become increasingly important for digitally interfaced experiments and underpin many of the applications discussed below in §4 and 5.

The integrated nature of a micro-LED array bonded to bespoke CMOS control electronics, combined with digital control through FPGA devices, provides a compact optical transmitter system capable of high-speed spatial and temporal modulation of optical signals. An optimized system could be developed on the scale of a few cm^2^, with a power consumption of less than 1 W and very low mass on the order of a few 100’s of grams [34]. This allows the multi-functional OWC systems described in this paper to be implemented in application areas with SWaP limitations, such as small satellites, drones, IoT devices and wearable technology.

## 4. Micro-light-emitting diode enabled optical wireless communication

As the bottleneck for OWC systems is often the optical transmitter, the high modulation bandwidth exhibited by micro-LEDs enables high data rate transmission. A detailed discussion of modulation schemes, multiplexing techniques, and hardware that underpin Gb s^−1^ OWC is given by Rajbhandari *et al.* [4], which provides an extensive review of visible light communications using GaN devices, including micro-LEDs, up to 2016. It is worth noting, however, that since its publication single micro-LED pixels using OFDM have reached data rates of almost 10 Gb s^−1^ [35]. The OWC results discussed in this paper will instead focus on communications demonstrations that take explicit advantage of the micro-LED format, regardless of the achieved data rates.

### (a) Series-connected micro-light-emitting diode transmitters

The high modulation bandwidth nature of a micro-LED is an advantage for high data rate transmission, however, the reduced size also reduces total output power. In turn, this reduces the achievable signal-to-noise ratio at a receiver, which can limit the performance of an optical link. This is of particular importance for application areas where high levels of loss may be experienced in the optical channel, such as for underwater optical wireless communications (UOWC), where light is scattered and absorbed in water [36]. An obvious approach to increase transmitter power, particularly in arrayed devices, is to increase the number of pixels in use. However, to preserve the modulation bandwidth of single micro-LEDs operated in parallel, each pixel must have an individual set of drive electronics, which becomes impractical for complex modulation schemes, as discussed in §3a.

**Figure 5.**
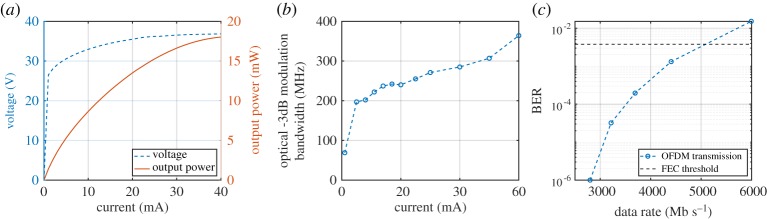
(*a*) Electro-optical performance of nine micro-LEDs connected in series. (*b*) Modulation bandwidth and (*c*) data rate performance using OFDM [18]. (Online version in colour.)

An alternative approach is to connect the pixels in series, as shown schematically in figure 1*e* [18]. By fully isolating micro-LED mesas down to the sapphire substrate, metal tracks can be deposited to interconnect LEDs from cathode to anode. Figure 5*a* shows the electro-optical performance of a series device composed of nine circular, 40 *μ*m diameter micro-LEDs. Naturally, the turn-on voltage for the device is increased by a factor of 9 over that of a single pixel due to the series connection. The device is able to produce 18 mW of optical power before thermal rollover, slightly less than nine times that of a single pixel due to heating effects and light absorption by neighbouring pixels. Importantly, figure 5*b* shows that the high modulation bandwidths are preserved, achieving over 280 MHz at 3200 A cm^−2^.

Achievable data rates were investigated when using the nine-element series device for free-space optical communications. Figure 5*c* shows the bit-error-ratio (BER) against data rate performance using OFDM. A BER below a forward error correction (FEC) threshold, indicated in figure 5*c*, can be considered ‘error free’ with application of an FEC algorithm. The BER curve intersects the FEC threshold at a data rate of 5.18 Gb s^−1^. Therefore, as the FEC algorithm introduces a 7% overhead, the error-free data rate achieved with the series device is 4.81 Gb s^−1^.

### (b) Multiple-input multiple-output transmitters

**Figure 6.**
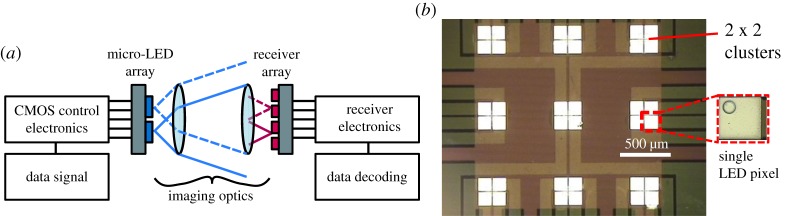
(*a*) Schematic of an imaging MIMO set-up. (*b*) Plan view optical micrograph of a micro-LED array for MIMO with a 3 × 3 set of 2 × 2 clusters of 39 *μ*m LED pixels. (Online version in colour.)

The performance of 4G and 5G RF mobile wireless networks is crucially underpinned by employing MIMO techniques to multiplex data over arrays of transmitters and de-multiplex them using an array of receivers. While the de-multiplexing of MIMO channels is generally a matrix inversion on the vector of received signals, adaptation to OWC enables an enhanced approach called ‘imaging MIMO’. In this case, optics at both transmitter and receiver arrays aid the channel separation process by physically separating the signals incident on the receiver array as indicated in figure 6. Micro-LEDs are ideally suited for this application, not only for their high bandwidth, but also because they can be fabricated into a low-density array of emitters that are spaced sufficiently apart, on the one hand, to allow efficient optical channel separation, yet on the other hand are sufficiently close to each other to remain in the field of view of standard lens optics. Up to 7 Gb s^−1^ were demonstrated using a 3 × 3 array of LEDs using one 39 *μ*m diameter pixel from each cluster of the device shown in figure 6*b* [32,37].

### (c) Digital to light conversion

**Figure 7.**
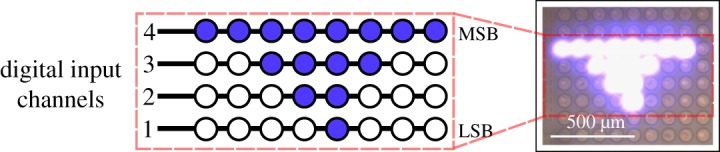
Schematic and plan view optical micrograph of a section from a 16 × 16 CMOS controlled micro-LED array with binary-weighted groups of pixels enabled. Applying digital signals to each column can produce 2^4^ discrete signals. (Online version in colour.)

In §3, the nonlinearity issues arising from the use of a single LED transmitter were highlighted, which may be exacerbated by nonlinearities of the DAC. This can seriously distort the transmitted waveforms and result in bit errors. However, with an LED array, discrete levels can be generated by activating binary-weighted groups of LEDs. With a CMOS-controlled micro-LED array, as in figure 7, this functionality is available on a millimetre-sized chip with digital control [38]. In effect, digital signals are directly converted to optical, removing the need for a DAC and avoiding the nonlinearity issues of a single pixel.

This approach has been used to demonstrate 4 and 8 level PAM with a symbol rate of 100 MHz [39]. In addition, analogue signals can be quantized into discrete levels, allowing the device to output OFDM signals despite being limited to on–off modulation of each pixel. This demonstrated a spectral efficiency of 3.96 bits s^−1^ Hz^−1^, though the data rate was limited by the maximum 100 MHz sampling rate of the FPGA [39].

### (d) Low single-photon avalanche diodes photon-counting communications

An important property of micro-LEDs is their small footprint, which even when combined with driver electronics can remain on a chip-scale level, with power consumption at or below the 1 W level. This makes them attractive for systems on mobile platforms, robots, underwater-vehicles, drones or nano-satellites. Many of these applications come with significant link distances, and the low overall optical output powers of micro-LEDs then require suitably sensitive detectors. This demand can be met by single-photon avalanche diodes (SPADs) [40], which combine photon-level sensitivity with a time resolution on the order of 100 ps. These devices offer low footprint, digital electronic interfacing, and spectral sensitivity which matches well with micro-LED emission.

**Figure 8.**
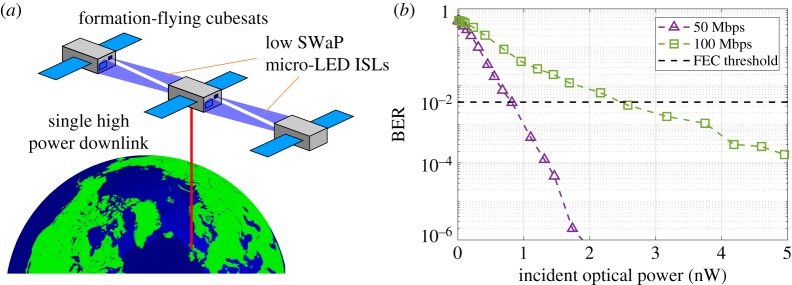
(*a*) Intersatellite links (ISLs) between formation flying CubeSats are provided by low SWaP micro-LED hardware. (*b*) Transmission results indicating low received optical power levels. (Online version in colour.)

Demonstrations using just a single micro-LED transmitter and SPAD-based receivers indicated that data rates of up to 100 Mb s^−1^ can be achieved with very low received power levels, while meeting the power constraints of typical nano-satellite platforms [34,41]. A potential application scenario is shown in figure 8*a*, where a network of CubeSats could communicate with low SWaP micro-LED systems, with only one unit requiring a higher power downlink system. Figure 8*b* shows the required received power levels are low, and ray-tracing simulations indicate these levels can be maintained over multi-kilometre ranges with simple optics [42].

## 5. Structured light capabilities linking imaging, tracking and communications

Driven initially by the prospect of high-brightness micro-displays, GaN micro-LEDs have been fabricated into high-density arrays with active-matrix control. The prospect of using this high degree of spatial diversity for OWC was exploited soon after creation of these devices, and preliminary results indicated that Gb/s rates should be attainable [43]. Since then, there have been technology demonstrations highlighting an array of novel capabilities of active-matrix micro-displays. In this section, we provide a review of the early activities in this emerging field.

### (a) Mobile phone camera communications through spatial patterns

Optical camera communications (OCC) is an attractive method for data transfer due to the widespread availability of high-resolution camera systems in portable consumer products, such as smartphones and laptops [44]. Although any device with an image sensor could receive an OWC signal, a common problem is the frame rate of the camera, typically 30 or 60 fps, which strongly limits the sampling rate of a signal and therefore data rate. However, smartphones are increasingly being produced with slow-motion camera systems with frame rates up to 240 fps, and some up to 960 fps. This can lift some of the data rate ceilings of OCC methods, though for maximum performance it is important for the system to fully exploit both spatial and temporal domains as much as possible.

**Figure 9.**
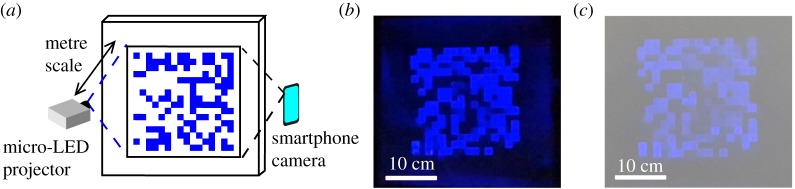
(*a*) Schematic of the OCC system using a micro-LED projector and smartphone camera. Example pseudorandom frames displayed on a wall under (*b*) dark conditions and (*c*) normal room lighting. (Online version in colour.)

A micro-LED array with suitable optics can be operated as a projector system, displaying structured light patterns on a surface [45]. With this approach, shown schematically in figure 9*a*, OWC can be achieved through the patterns imaged by a smartphone camera. Each pixel in the micro-LED array transmits an independent on–off keying (OOK) data stream, resulting in patterns that appear pseudorandom, as shown in figures 9*b*,*c*. Data rates can be scaled in the spatial domain, by adding pixels and therefore parallel streams, or temporal domain, by increasing the frame rates of the camera and projector. Proof-of-concept experiments have shown a data rate of 122.88 kb s^−1^ with a 16 × 16 array of micro-LEDs and a Samsung Galaxy S9 smartphone [45].

The high-speed nature of micro-LED display systems allows the transmitter in this OCC implementation to readily keep pace with the frame rates emerging in consumer camera technology. With a moderate increase in transmitter resolution, data rates scale to Mb s^−1^ rates. For example, using the same resolution as a version 19 QR code (93 × 93), a data rate of 4.15 Mb s^−1^ could be achieved. In contrast, applying the high frame rate camera to alternative transmitters for spatially encoded OCC results in limited scaling due to transmitter frame rate limitations, whereas the micro-LED systems are limited by the receiver frame rates.

### (b) Data through display

**Figure 10.**
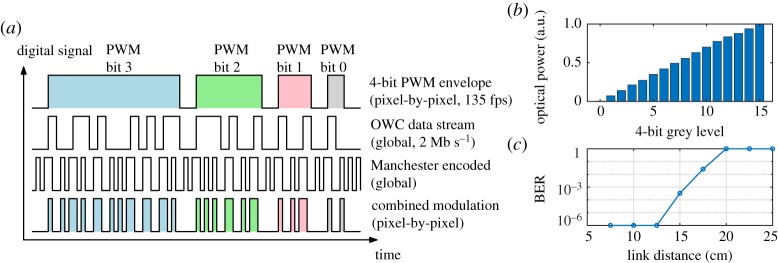
Data through display: (*a*) modulation scheme with pixel-wise greyscale video display using PWM, overlayed with a global OWC data signal using Manchester-encoded OOK. (*b*) Normalized optical power of one pixel for 16 levels, data from [23]. (*c*) BER as a function of distance for 2 Mb s^−1^, simultaneously displaying a video at 135 fps, data from [47]. (Online version in colour.)

Li-Fi technology relies on the dual use of LEDs for lighting and OWC at the same time. Similarly, micro-LED displays could be made to display content to human users while at the same time transmitting data through an OWC signal that can be sensed by a photodetector. In a first instance, the display could encode the data in frames that are displayed at the standard video frame rate (e.g. 30 fps). This approach was indeed used for high data rate camera communications [46], however, during the data transfer the normal display function is disabled or compromised. The display function would be retained if the display was operated at a high frame rate (kfps or more) and data encoded such that within visually recognizable timescales (about 50 ms) the value of each pixel averages to the greyscale value of the currently displayed image. It would be challenging to do this through individual pixel addressing due to the high data rates required, however, simpler alternatives are possible. A global or row-wise OWC data signal can be overlayed at pixel level with the greyscale setting, such that the correct grey level of the pixel is preserved and the OWC signal is transmitted at a speed invisible to the eye. Li *et al.* [23,47] implemented such a configuration by overlaying PWM for brightness control at 135 fps with a global Manchester-encoded OOK OWC signal at 2 Mb s^−1^, as illustrated in figure 10. This approach has since been applied to OCC at a peak rate of 16.6 kb s^−1^ and an average rate of 5 kb s^−1^ [48].

### (c) Visible light positioning through structured illumination

An active area of research related to OWC is in positioning systems. While the global positioning system (GPS) is the dominant technology for self-location across the globe, it has drawbacks in indoor environments, and areas where line-of-sight to the satellites may be blocked. Instead, optical signals from light fittings can be used as local navigation systems, providing high positioning accuracy, and brings similar advantages to optical communications such as no RF interference and integration with SSL [49]. In addition, positioning functionality can be employed in parallel with communication functions, enhancing communication performance by enabling space-division multiple access data transmission and assisting handover between transmitters, important functions for Li-Fi style systems [3,50].

**Figure 11.**
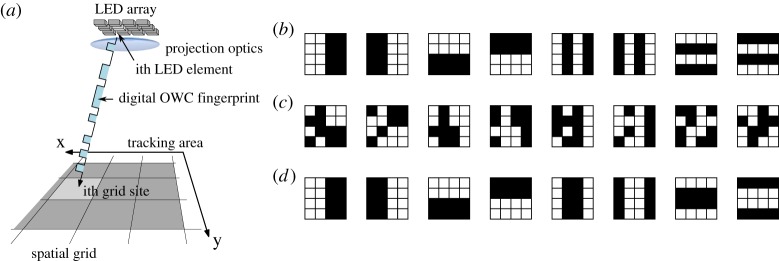
(*a*) Schematic of positioning using digital fingerprints by structured illumination from a micro-LED array. Example pattern sequences for fingerprinting with a 4 × 4 array using (*b*) binary search, (*c*) pseudorandom and (*d*) moving bars patterns. (Online version in colour.)

There are many approaches to visible light positioning (VLP), including triangulation, time-of-arrival and probabilistic methods. Structured illumination provided by micro-LED arrays can be used to determine position based on fingerprint signals supplied to a spatial grid [50,51]. The concept is shown schematically in figure 11*a*, where a micro-LED array is imaged onto a tracking area, with each pixel illuminating a spatially separated area. By careful choice of binary illumination patterns, each area receives a unique set of on-off signals, from which position can be determined. There are many potential binary pattern sequences, with binary search patterns, pseudorandom patterns and ‘moving bars’ patterns shown in figure 11*b*,*c*,*d* respectively, for an example 4 × 4 array. Larger arrays require more patterns to generate unique signals for each pixel. The advantages of this approach are low computational complexity, low receiver hardware requirements, and scalability through appropriate optics. Indeed, scaling down to a microscopic level for integration into a direct-writing optical lithography tool has recently been shown [52], and scaling to kilometre link distances is believed to be possible with a suitably sensitive receiver [41]. Flicker-free operation is guaranteed by update rates of up to 30 kfps [53].

## 6. Conclusion and prospects

Micro-LED arrays provide a high-bandwidth technology for many forms of OWC with sophisticated control of the spatial characteristics of the transmitted signal. They are able to meet tight SWaP constraints and can be conveniently interfaced with digital electronics, providing source technology for the emerging application area of structured lighting, which combines high-density spatial encoding with the LEDs’ fast switching characteristics. Micro-LEDs are well matched with SPAD receivers, which also offer low SWaP and digital electronic interfacing. Exploiting photon-sensitive detection, micro-LEDs can be used for OWC in challenging environments, e.g. underwater or in space. With ongoing developments of hardware and electronic driving schemes, micro-LEDs are capable contenders for many IoT related applications and are well placed to foster emerging convergences between lighting, display and communications.

## Data accessibility

This article does not contain any additional data.
